# Genetic, Cellular and Molecular Aspects involved in Apical Periodontitis

**DOI:** 10.1590/0103-6440202205113

**Published:** 2022-08-26

**Authors:** Igor Bassi Ferreira Petean, Alice Corrêa Silva-Sousa, Tamara Justiniano Cronenbold, Jardel Francisco Mazzi-Chaves, Lea Assed Bezerra da Silva, Raquel Assed Bezerra Segato, Guilherme Assed Piedade de Castro, Erika Calvano Kuchler, Francisco Wanderley Garcia Paula-Silva, Manoel Damião Sousa-Neto

**Affiliations:** 1 Department of Restorative Dentistry, School of Dentistry of Ribeirão Preto, University of São Paulo, Brazil.; 2 Department of Pediatric Dentistry, School of Dentistry of Ribeirão Preto, University of São Paulo, Ribeirão Preto, São Paulo, Brazil.; 3 Department of Orthodontics, University of Regensburg, Franz-Josef-Strauss-Allee 11, 93053 Regensburg, Germany.

**Keywords:** Pulp biology, apical periodontitis, bone remodeling process, host response, genetics

## Abstract

The development, establishment and repair of apical periodontitis (AP) is dependent of several factors, which include host susceptibility, microbial infection, immune response, quality of root canal treatment and organism's ability to repair. The understanding of genetic contributions to the risk of developing AP and presenting persistent AP has been extensively explored in modern Endodontics. Thus, this article aims to provide a review of the literature regarding the biochemical mediators involved in immune response signaling, osteoclastogenesis and bone neoformation, as the genetic components involved in the development and repair of AP. A narrative review of the literature was performed through a PUBMED/MEDLINE search and a hand search of the major AP textbooks. The knowledge regarding the cells, receptors and molecules involved in the host's immune-inflammatory response during the progression of AP added to the knowledge of bone biology allows the identification of factors inherent to the host that can interfere both in the progression and in the repair of these lesions. The main outcomes of studies evaluated in the review that investigated the correlation between genetic polymorphisms and AP in the last five years, demonstrate that genetic factors of the individual are involved in the success of root canal treatment. The discussion of this review gives subsides that may help to glimpse the development of new therapies based on the identification of therapeutic targets and the development of materials and techniques aimed at acting at the molecular level for clinical, radiographic and histological success of root canal treatment.

## Resumo

O desenvolvimento, estabelecimento e reparo da periodontite apical (PA) depende de vários fatores, que incluem a susceptibilidade do hospedeiro, infecção microbiana, resposta imune, bem como a qualidade do tratamento do canal radicular e a capacidade de reparo do organismo. A compreensão das contribuições genéticas para o risco de desenvolver a PA e apresentar PA persistente tem sido extensivamente explorada na Endodontia moderna. Assim, este manuscrito pretende fornecer uma revisão da literatura em relação aos mediadores bioquímicos envolvidos na sinalização da resposta imune, osteoclastogênese e neoformação óssea, bem como os componentes genéticos envolvidos no desenvolvimento e reparo da PA. Uma revisão narrativa da literatura foi realizada através de uma pesquisa nas bases PUBMED/MEDLINE e uma pesquisa manual nos principais livros sobre a PA. O conhecimento sobre as células, receptores e moléculas envolvidas na resposta imuno-inflamatória do hospedeiro durante a progressão da PA somado ao conhecimento da biologia óssea, especialmente o papel dos osteoblastos, osteócitos e osteoclastos no turnover ósseo, permite a identificação de fatores inerentes ao hospedeiro que podem interferir tanto na progressão como no reparo destas lesões. Os principais resultados dos estudos avaliados na revisão que investigaram a correlação entre polimorfismos genéticos e PA, nos últimos cinco anos, demonstram que os fatores genéticos do indivíduo estão envolvidos no sucesso do tratamento do canal radicular. A discussão desta revisão fornece subsídios que podem ajudar a vislumbrar o desenvolvimento de novas terapias baseadas na identificação de alvos terapêuticos e no desenvolvimento de materiais e técnicas destinadas a atuar a nível molecular para o sucesso clínico, radiográfico e histológico do tratamento endodôntico.

## Introduction

When microorganisms infect dental mineralized tissues, the components of the immune system recognize these invading microorganisms as non-self ^1^, consequently an inflammatory process is established in the pulp tissue, which results in the recruitment of chronic inflammatory cells, that involves the recruitment and activation of different cell types to fight the infection. In cases of this stimulus remains persistent, the pulp tissue evolves to necrosis, with subsequent resorption of mineralized tissues, resulting in the formation of a periapical osteolytic lesion ^2^.

Root canal treatment aims to eliminate the infection of the root canal system (RCS), through the neutralization of the aggressive agents of bacterial origin, by use of an adequate biomechanical preparation protocol, including the use of irrigating solutions and intracanal medication ^3^ ^,^ ^4^. Thus, after the cleaning and shaping of root canals, the disinfection of the RCS is reached, and tridimensional filling should be performed, in order to provide the repair of periapical tissues and success of the treatment.

However, the presence of apical periodontitis (AP) is associated with a higher failure rate after root canal treatment ^5^ ^,^ ^6^ ^,^ ^7^ ^,^ ^8^. This rate is dependent on different factors, once the anatomic complexity of the RCS, in addition to the location of the apical foramen, may hinder the complete disinfection ^4^ ^,^ ^7^ ^,^ ^9^. Moreover, the host immune system plays an important role in trying to eliminate the aggressive agent present in areas inaccessible to biomechanical preparation.

In some situations, it is possible to eliminate the aggressor agent by means of the immune and inflammatory responses triggered to neutralize/destroy these agents, resulting in the resolution of AP ^10^. However, in other situations, due to unsatisfactory host defense to infection present in root canals ^11^,^12^, or following exacerbated and persistent activation of the host innate and adaptive immune system, AP remains persistent to root canal treatment ^13^. Yet, there are several host genetic components involved in the establishment, progression and repair of AP ^14^. Thus, this article aims to provide a review of the literature regarding these components associated with the biochemical mediators involved in the signaling in the immune response, osteoclastogenesis and bone neoformation, during the development and repair of AP.

## Influence of genetics on apical periodontitis

Considering the differences in the human DNA sequence, not all individuals have the same response to a certain stimulus or treatment due to differences in the human DNA sequence, which influence the organism's susceptibility to disease and its responses to the environment ^15^ ^,^ ^16^. These variations are considered normal but when found in more than 1% of the population are called genetic polymorphisms ^15^ ^,^ ^17^ ^,^ ^18^ ^,^ ^19^.

When there is a substitution of one nucleotide for another, occurring the exchange of a base pair, they are called single nucleotide polymorphisms (SNPs), which are the most common type of polymorphism. This exchange can still affect protein expression, structure and function of a gene ^20^. Thus, genetic variations caused by mutations or genetic polymorphisms may influence host response.

Both genetic polymorphisms and mutations can be located in various regions of the gene, such as the promoter region, coding region (exons) and non-coding region (introns). In general, genetic variations in the promoter and coding region are more likely to modify the function of the gene and consequently alter protein formation. Changes in the coding region may, for example, lead to an amino acid substitution in the protein sequence, which may cause structural and functional modifications in the protein and a potential biological effect. Thus, genetic polymorphisms can alter protein synthesis and cellular function, which may affect the progression of AP ^20^ ^,^ ^22^ ^,^ ^23^.

Considering that, AP is a multifactorial disease ^24^ from the polymicrobial origin and represents a localized immunoinflammatory response, characterized by the presence of a mixed inflammatory infiltrate ^25^ ^,^ ^26^ ^,^ ^27^ ^,^ ^28^ ^,^ ^29^, the investigation of the interaction between molecular signals, genetic influence and clinical signs of AP is a promisor topic of research.

**Figure 1:**
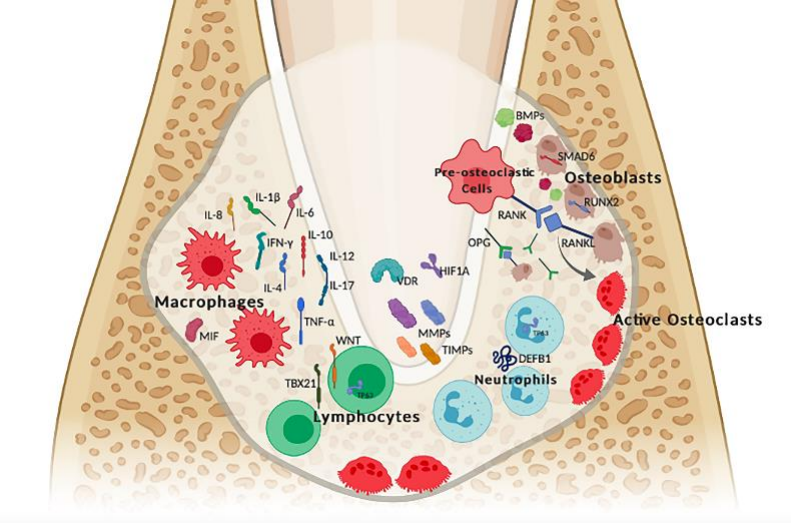
Representation of cellular, molecular components and mediators linked to inflammation and bone metabolism processes that were evaluated regarding the influence of genetic polymorphisms in the development, progression and repair of apical periodontitis. Cytokines (IL-1β, -4, -6, -8, -10, -12, -17, TNF-α, INF-γ) and macrophage migration inhibitory factor (MIF) are intimately related to the macrophage’s activity. RANK, RANKL, OPG are main controllers of the bone metabolism process. RUNX2, SMAD6 are transcriptional factors of osteoblast activity. BMPs, MMPs, TIMPs are proteins secreted into the extracellular media. DEFB1 is a defensine secreted by neutrophils. HIF1A gene encodes the alpha subunit of transcription factor HIF-1a, which regulates oxygen dependent gene transcription. VDR is recognized as a member of the super-family of nuclear receptors that regulate genes expression and has a central role in the biology of vitamin D action. WNT, TBX21 and TP63 comprehends respectively, signaling pathway, a gene involved in the activity of lymphocytes, a gene that encodes a TP63 protein, which controls the cell activity.

In recent years, interactions between genetic polymorphisms and the development, progression and repair of AP have been evidenced in genes linked to inflammation and bone metabolism processes ^23^ ^,^ ^30^ ^,^ ^31^ ^,^ ^32^ ^,^ ^33^ ^,^ ^34^ ^,^ ^35^ ^,^ ^36^ ^,^ ^37^ ^,^ ^38^ ^,^ ^39^ ^,^ ^40^ ^,^ ^41^ ^,^ ^42^ ^,^ ^43^ ^,^ ^44^ ^,^ ^45^ ^,^ ^46^ ^,^ ^47^ ^,^ ^48^ ^,^ ^49^ ^,^ ^50^, which are represented at Figure 1. Thus, there is an important discussion of these molecular aspects involved in the etiopathogenesis of dental caries, AP and repair after root canal treatment, which will be addressed throughout the next topics.

### Cellular and molecular components on apical periodontitis development

The localized, destructive and progressive infection of mineralized tissues of the tooth, dental caries, when untreated can result in implications for the dental pulp ^13^, which is a specialized connective tissue, richly vascularized and innervated, of ectomesenchymal origin, that presents sensory, protective, inductive, formative and nutritive functions ^51^.

In response to infection in the dental pulp and periapical tissues, activation of innate immune system cells occurs locally ^52^. The cells of the innate immune system possess receptors that recognize pathogen-associated molecular patterns (PAMPs), which includes bacterial components such as lipopolysaccharide (LPS) and lipoteichoic acid (LTA) ^53^. Through this pattern recognition receptors (PRRs), among which Toll-like receptors (TLRs) are prominent, cells are able to respond to pathogen invasion. TLRs are type I transmembrane proteins ^54^ and have been identified, in humans, expressing TLRs 1 to 10 and, in mice, expressing TLRs 1 to 9 and 11 to 13 ^55^.

After the activation of pathogen recognition receptors and the expression of intra- and extracellular biochemical mediators, the activation of different cell types occurs. As part of the immune-inflammatory response, neutrophils, macrophages, lymphocytes, plasma cells, dendritic cells and natural killer cells are recruited to the site of inflammation in order to eliminate the aggressor agent ^2^ ^,^ ^24^ ^,^ ^25^ ^,^ ^56^. This action is coordinated and regulated by the release of cytokines, chemokines, growth factors, extracellular matrix components and other bioactive molecules ^2^ ^,^ ^27^ ^,^ ^57^ ^,^ ^58^.

Initially, upon encountering pathogens, tissue macrophages stimulate an inflammatory process via the release of cytokines and chemokines, resulting in the recruitment and activation of other cells, such as neutrophils and monocytes ^59^ ^,^ ^60^. Macrophages are phagocytic cells of innate immunity and, together with neutrophils, provide the first line of defense against the microorganism ^59^. Macrophages attempt to prevent the invasion of these agents through phagocytosis, secretion of lytic enzymes and activation of the complement system ^59^ ^,^ ^60^. In addition to their phagocytic function, macrophages act as antigen-presenting cells to lymphocytes, mediating adaptive immunity ^56^.

With the progression of AP, there is an infiltration of cellular elements of adaptive immunity, including the participation of B and T lymphocytes ^2^ ^,^ ^27^ ^,^ ^52^. Lymphocytes, which express antigen-specific receptors, represent the key cells of the adaptive immune system and originate from bone marrow precursors to differentiate into mature effector cells in the periphery. B-lymphocytes recognize antigen by their cellular receptor BCR and produce different antibodies. T lymphocytes have the TCR receptor that recognizes antigen by means of molecules expressed on the cell surface, known as major histocompatibility complex.

In addition to cellular recruitment, during the inflammatory response, several biochemical mediators are released locally with the aim of stimulating the cellular and humoral immune response. Among these inflammatory mediators are the eicosanoids, which are synthesized from the metabolism of arachidonic acid, produced by the action of phospholipase enzymes. Through the action of cyclooxygenases (COX) or lipoxygenases (LO) enzymes, structural modifications occur in the arachidonic acid chain, leading to the synthesis of prostaglandins and thromboxanes or leukotrienes and lipoxins, respectively ^61^ ^,^ ^62^. Prostaglandins increase local blood flow, vascular permeability and edema formation, and amplify the pattern of the inflammatory response, to promote both the increase and prolongation of the effects and signals produced by pro-inflammatory agents ^61^. Leukotrienes have important biological functions, including an efficient chemotactic action, aggregation and degranulation of polymorphonuclear cells, as well as stimulating leukocyte adherence to the endothelial wall for transmigration of inflammatory cells ^63^ ^,^ ^64^.

Pro- and anti-inflammatory cytokines are also important signalers for host defense. Cytokines function as messengers and represent a family of glycoproteins that coordinate biological processes such as embryonic development, immunity, hematopoiesis and repair ^65^ ^,^ ^66^ ^,^ ^67^. The pro-inflammatory cytokines include the interleukins (IL) -1α, -1β, -6, -8, and the tumor necrosis factor-alpha (TNF-α), among others ^68^. Interleukins, particularly IL-1α and IL-1β, are produced in AP by different cell types including macrophages, osteoclasts, polymorphonuclear cells and fibroblasts ^69^ ^,^ ^70^. The local effects of IL-1β consist of increased leukocyte adhesion to endothelial walls, stimulation of lymphocytes, potentiation of neutrophils, production of prostaglandins and proteolytic enzymes, increased bone resorption and inhibition of bone formation ^71^ ^,^ ^72^. IL-6 is produced under the influence of IL-1β, TNF-α and interferon-γ (INF-γ), acting as a negative regulator of production and antagonizing the effects of IL-1β ^73^. The production of IL-8 is carried out by monocytes, macrophages and fibroblasts under the influence of IL-1β and TNF-α ^74^, which, being a chemo-attractant, is of paramount importance in the acute phase of AP, in which a massive infiltration of neutrophils occurs ^25^.

IL-22 is a cytokine belonging to the IL-10 family ^75^ and expressed by different types of lymphocytes from both the innate and adaptive immune systems. This includes CD4 T cell subsets, most notably Th17 cells ^76^ ^,^ ^77^. In addition, this is the main effector cytokine of Th22 helper T cells ^78^. IL-22 contributes to the expression of several molecules encoding genes involved in the inflammatory response including IL-6, G-CSF (granulocyte colony-stimulating factor) and IL-1α ^79^ ^,^ ^80^. This cytokine has been shown to act on hepatocytes, epithelial cells, keratinocytes and fibroblasts, inducing in vitro and in vivo an acute phase response and stimulation of the release of chemokines and matrix metalloproteinases ^81^ ^,^ ^82^ ^,^ ^83^.

Cytokines act on different signaling networks and it has been described that TNF- α, IL-17, IFN-γ and IL-1β influence some effects of IL-22 ^79^. It is worth noting that T cells differentiate towards the Th22 phenotype in the presence of some cytokines, such as TNF-α and IL-6 ^84^ and, in the presence of IL-1β and IL-6, differentiate towards the Th17 phenotype ^77^. The dual nature of this response, sometimes synergistic and sometimes antagonistic, played by IL-22 is related to the inflammatory context, which includes the duration and accumulation of the cytokine, the global cytokine milieu and the type of tissue involved ^76^. IL-22 may also act synergistically with several other cytokines, including IL-17A, IL-17B and TNF-α ^77^ ^,^ ^85^. Co-secretion of IL-22 with pro-inflammatory agents such as TNF-α, IFN-γ and/or IL-17 results in a significant increase in the immune-inflammatory reaction, whereas IL-22 alone has a protective and regenerative effect ^86^. For the above, in experimental AP, it was demonstrated that IL-22 clearly modifies the pattern of the inflammatory response and the absence of this cytokine resulted in a smaller extension of lesions and a reduction in the number of osteoclasts, especially in late periods when a chronic inflammatory infiltrate is prevalent ^87^.

As a way of controlling the immune response, the host cells have a mechanism to inhibit the exacerbated production of pro-inflammatory cytokines. This process happens by cytokine signaling suppressor proteins called SOCS-1, SOCS-2 and SOCS-3 ^65^ ^,^ ^66^ ^,^ ^88^ ^,^ ^89^. SOCS-1 is activated by the presence of INF-γ, TNF-α, IL-6, or even by exposure to bacterial lipopolysaccharide, and its action occurs by inhibition of the expression of the same INF-γ, TNF-α and IL-6 ^65^ ^,^ ^66^ ^,^ ^88^. SOCS-3, in general, acts on the expression of IL-1, IL-6, IL-10 and INF-γ ^65^ ^,^ ^66^ and has been found in periapical granulomas, together with the expression of IL-10 ^89^. Added to this, it is known that SOCS-3 expression is induced by pro-inflammatory cytokines and this condition inhibits the secretion of chemokines induced by IL-1β or IL-6. SOCS-3 protein expression in humans plays important negative feedback, suppressing AP progression ^90^. These proteins have been found in bone diseases ^91^, periodontal disease ^92^ ^,^ ^93^ and AP ^89^ ^,^ ^90^ ^,^ ^94^, suggesting an important defense mechanism of the body in combating exacerbated inflammation and bone loss.

## Mediators involved in the resorption of mineralized tissues

The main players involved in the resorption process of bone and tooth structures are known as canonical mediators of osteoclastogenesis and include the nuclear factor activating receptor NF-kB (RANK), RANK ligand (RANKL) and osteoprotegerin (OPG) ^95^ ^,^ ^96^. RANK is a receptor found on the surface of clastic cells acting in cell differentiation. RANKL is a soluble ligand synthesized by osteoblasts and osteocytes and cells of the immune system and, when bound to the RANK receptor, induces the expression of genes that specify the osteoclast lineage such as the enzyme tartrate-resistant acid phosphatase (TRAP), matrix metalloproteinase-9 (MMP-9), cathepsin K and the receptor for calcitonin, as well as differentiation, maturation and activation of osteoclasts to stimulate resorption of mineralized tissues ^95^ ^,^ ^97^. RANKL induces osteoclast maturation and activity ^95^. OPG, on the other hand, is a soluble receptor binds to RANKL and inhibits osteoclast differentiation and activity. The imbalance in RANKL and OPG expression in inflammatory conditions, in which there is increased RANKL and decreased OPG activity, results in exacerbated osteoclastogenesis and bone resorption ^2^ ^,^ ^89^ ^,^ ^98^.

The cellular component of bone tissue is comprised of osteoblasts, surface or lining cells (lining cells), osteoclasts and osteocytes. Among these cell types, osteocytes represent more than 95% of the cells present and increase in number according to the age and size of the bone ^99^ ^,^ ^100^, while osteoblasts correspond to less than 5% and osteoclasts to less than 1% ^101^. Osteocytes remain viable for decades, whereas osteoblasts survive for weeks and osteoclasts for days ^99^ ^,^ ^102^.

Thus, another important mechanism in the process of bone remodeling occurs via signaling by osteocytes. These cells participate in bone formation through the expression of proteins such as type I collagen and osteocalcin in addition to proteins involved in the mineralization of this tissue such as alkaline phosphatase ^103^. Moreover, the effect of osteocytes on bone formation is related to their interference in the Wnt/β-catenin complex, one of the most important signaling pathways responsible for the regulation of osteoblast function ^103^ ^,^ ^104^.

In root canal infection, microorganisms and their by-products stimulate the local inflammatory response and intense production of proteases that degrade the extracellular matrix and facilitate the resorption process of mineralized tissues, both bone and teeth ^105^ ^,^ ^106^ ^,^ ^107^. Among the proteases are the matrix metalloproteinases (MMPs), an important family of metallopeptidases, capable of degrading components of the extracellular matrix (ECM), including the organic portion of the bone. Members of the MMP family are divided into collagenases (MMP-1, -8 and -13), gelatinases (MMP-2 and -9), stromelysins (MMP-3 and -10), membrane MMPs (MMP-14, -15, -16, -17 and -24) and others (MMP-7, -12, -19, -20, -21, -22 and -23) ^108^. MMPs are synthesised as latent enzymes that may be present inside inflammatory cells, but are most often membrane-bound or embedded in the extracellular matrix ^109^ ^,^ ^110^.

In parallel to the disorganization of the collagen matrix in AP, resorption of inorganic mineralized tissue also occurs from osteoclastogenesis. In this process, monocytes coming from the circulation are recruited by macrophage colony-stimulating factor (M-CSF), fuse and form osteoclasts, differentiated cells specialized in the process of degradation of bone matrix components ^111^ ^,^ ^112^. Osteoclasts, characterized as multinucleated giant cells, present in their membrane calcitonin receptor, and are positive for the enzyme tartrate-resistant acid phosphatase (TRAP) ^111^ ^,^ ^112^ ^,^ ^113^.

The immune response generated against bacteria or by-products, although it is a defense response, may lead to different degrees of injury to the organism. In inflammation near bone tissue, for example, there is a close relationship between the mediators of the inflammatory response and the metabolism of mineralized tissue. In periapical injury, pro-inflammatory cytokines stimulate the resorption process and inhibit bone neoformation ^2^ ^,^ ^114^. On the other hand, anti-inflammatory cytokines are responsible for coordinating the activity of inflammatory cells aiming at repair. In vivo studies with genetically modified animals deficient in different receptors and cytokines confirm the relationship between the expression of anti- and pro-inflammatory cytokines associated with AP progression and osteoclastogenesis induction ^87^ ^,^ ^115^ ^,^ ^116^.

The increased synthesis of prostaglandin E2 in teeth with periapical inflammation is related to inflammatory and catabolic (pro-resorptive) changes that occur in AP ^117^ and the decreased production of this lipid mediator may be an indication of disease remission ^118^. TNF-⍺ plays an important role in the inflammatory response of pulp tissue and AP development ^119^ ^,^ ^120^. It is a cytokine stimulated by monocytes/macrophages, polymorphonuclear neutrophils and fibroblasts ^25^ and has the ability to stimulate a group of cytokines called chemokines, whose chemotactic action assists in fighting the infectious process ^121^. On the other hand, TNF-⍺ regulates the synthesis of bone matrix protein, increases the production of interleukin-6 and macrophage stimulating factor (M-CSF) by osteoblasts, and indirectly promotes the differentiation of osteoclasts ^122^. In an animal model, gene expression analysis showed a positive correlation between MyD88-RANKL and TLR2-MyD88 expression, indicating the relevance of the immune response in bone loss arising from intra-canal bacterial infection ^123^.

Similarly, on the tooth surface, during the process of resorption of dentin and cementum, the presence of dentinoclasts and cementoclasts from the monocytic lineage is observed and present functions similar to those of osteoclasts ^121^ ^,^ ^123^. This group of cells are collectively known as clasts because they exert similar physiological or pathological activities according to the tissue they absorb, since there is no structural, organizational and functional difference between these cells ^112^.

## Influence of genetic polymorphisms on apical periodontitis repair: data from literature

The persistence of AP, after root canal treatment, may be related to different factors, including aspects of the quality of root canal treatment, such as presence of root perforations, instrument fractures and quality of root canal filling, as well as the non-effective removal of microorganisms and their by-products from the RCS, including the external root surface in the form of biofilm ^124^. It is also noteworthy that, in addition of aspects of the biomechanical preparation, the host response against the pathogenic potential of microorganisms and their susceptibilities to antimicrobials commonly used in Endodontics has been studied as one of the factors that define success or failure after root canal treatment ^28125^ ^,^ ^126^ ^,^ ^127^ ^,^ ^127^.

In recent years, understanding the genetic contributions to the risk of developing AP and the risk of presenting persistent AP has been investigated ^16^ ^,^ ^30^ ^,^ ^31^ ^,^ ^32^ ^,^ ^33^ ^,^ ^34^ ^,^ ^35^ ^,^ ^36^ ^,^ ^37^ ^,^ ^38^ ^,^ ^39^ ^,^ ^40^ ^,^ ^41^ ^,^ ^42^ ^,^ ^43^ ^,^ ^44^ ^,^ ^45^ ^,^ ^46^ ^,^ ^47^ ^,^ ^48^ ^,^ ^49^ ^,^ ^50^, since genetic polymorphisms may be biological modifiers of individual susceptibility in the development and course of diseases, including AP ^12^. AP is a multifactorial disease, in which some factors such as genetic polymorphisms and epigenetic factors could be involved in persistent AP after root canal treatment ^128^. Considering its multifactorial characteristics, this disease must be treated with an approach to the microbiology, quality of treatment and host response particularities ^41^.

**Figure 2:**
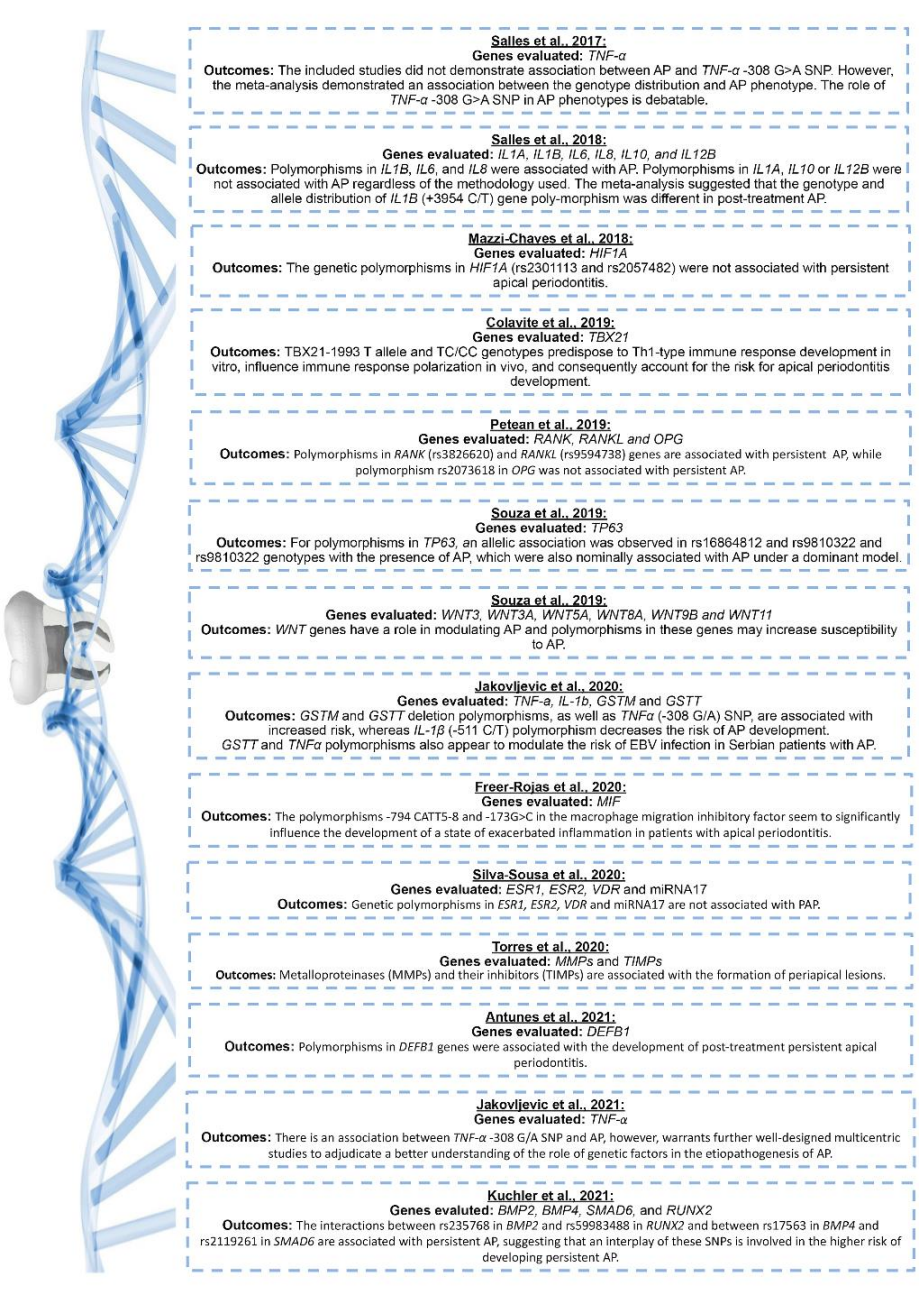
Studies that investigated the correlation between genetic polymorphisms and apical periodontitis, in the last five years: genes evaluated and main outcomes.

From this, we performed a review of the literature, regarding the influence of genetic components on AP. The search strategy included the terms “genetic polymorphisms” AND “apical periodontitis”, was performed in October 2021, on PUBMED/MEDLINE database, and only studies written in English were selected. The inclusion criteria include studies that evaluated the association between genetic polymorphisms with the development, persistence and clinical signs and symptoms of AP, performed in the last five years. From a total of 46 studies, 14 were included and their main outcomes were represented in Figure 2.

The differences in the outcomes obtained by these studies, is explained by the fact that functional effects of genetic polymorphisms are dependent of the region of the gene that occurs the exchange of a base pair ^15^ ^,^ ^20^ ^,^ ^21^ ^,^ ^22^. The SNPs in the coding region of gene are divided into two types: synonymous and nonsynonymous SNPs. The synonymous SNPs do not change the amino acid sequence of protein or not affect the protein function. The nonsynonymous SNPs are divided into two types: missense and nonsense. A missense SNP, arise in the coding region that alters the amino acid configuration which may have impact on structure and function of protein ^129^. For nonsense, a point mutation in a sequence of DNA that changes to a stop codon results in a nonfunctional protein product. Besides that, SNPs that are in non-coding regions of gene or in the intergenic regions may affect gene splicing (SNPs at intron region), transcription factor binding (SNPs at 5′ untranslated region), messenger RNA degradation, or the sequence of non-coding RNA. The type of SNPs located upstream or downstream from the gene that affects gene expression is referred to an expression SNP (eSNP). Thus, in the evaluated studies, while some genetic polymorphisms were associated with a higher risk to develop persistent AP, others have protective role.

It is noteworthy that these studies are still controversial regarding methodological and sampling variables, from the point of view of disease, treatment, and ethnic and individual factors ^14^ ^,^ ^130^. The pleiotropic effect of genetic polymorphisms may explain part of the associations observed in epidemiological studies between AP and different systemic alterations, such as metabolic syndrome ^131^ ^,^ ^132^, ischemic heart disease ^133^ and diabetes ^134^. Additionally, a recent review emphasized the need for further studies within new cohorts of different populations and ethnicities to either confirm or refute the role of genetic polymorphisms in AP ^135^. However, despite the controversies and the small number of studies that evaluated the role of genetic polymorphisms in the response of patients to root canal therapy, the existing results clearly demonstrate that genetic factors of the individual are involved in the success of root canal treatment. Further longitudinal studies are required to replicate the data obtained in these studies and to analyze gene expression, synthesis and protein activity of pro- and anti-inflammatory cytokines involved in the etiopathogenesis and repair of chronic AP, this may help to glimpse the development of new therapies, materials and techniques.

## Conclusions

Genetic polymorphisms in genes related to the host immune response, as well as genes involved in bone repair mechanisms, are involved in the individual's response to treatment and may in the future serve as biomarkers in clinical practice. Moreover, the knowledge regarding the cells, receptors and molecules involved in the host's immune-inflammatory response during the progression of AP added to the knowledge of bone biology, especially the role of osteoblasts, osteocytes and osteoclasts in bone turnover allows the identification of factors inherent to the host that can interfere both in the progression and in the repair of these lesions. This may help to glimpse the development of new therapies based on the identification of therapeutic targets and the development of materials and techniques aimed at acting at the molecular level for clinical, radiographic and histological success of root canal therapy.
